# Supplementary material for: An Alpha-Glucan from Lomentospora prolificans Mediates Fungal–Host Interaction Signaling through Dectin-1 and Mincle
Source: J Fungi (Basel). 2023 Feb 23;9(3):291. doi: 10.3390/jof9030291 (PMC10054066; doi:10.3390/jof9030291)
Supplement: Supplementary file 1 [file jof-09-00291-s001.zip › jof-2213736-supplementary.pdf]

Supplementary material

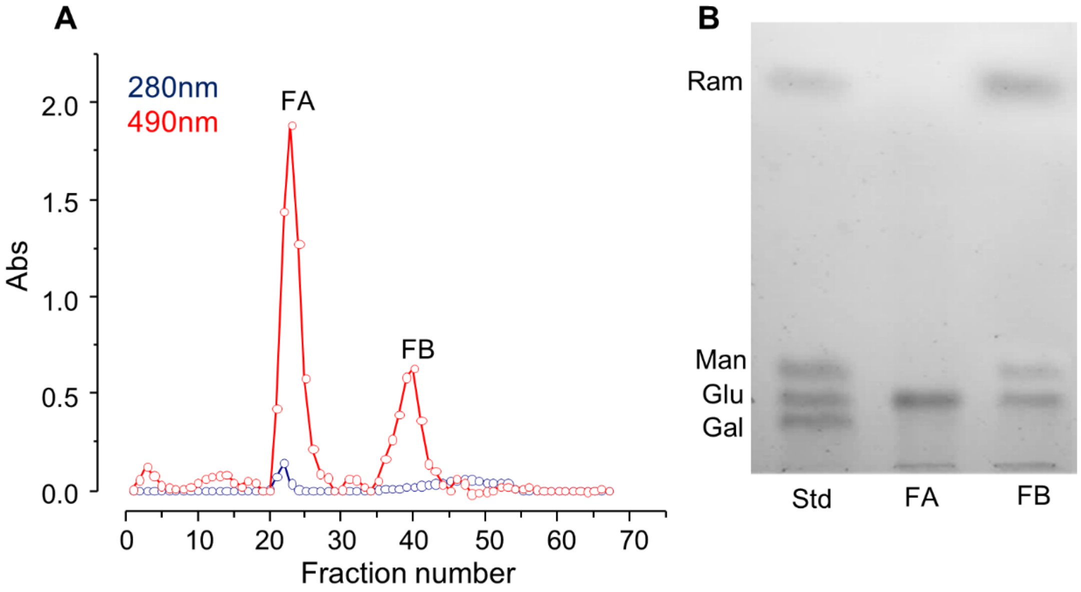

Figure S1. Purification of glucan from *Lomentospora prolificans*. (A) Elution profile of polysaccharides from *L. prolificans* on a Superdex 200 column with 0.15 M sodium chloride in 0.01M sodium phosphate, pH 7.0 as eluant. Eluted fractions were monitored by reading at A 280 nm for protein detection (blue line) and colorimetrically for carbohydrate (red line - A 490 nm) by the phenol-sulfuric acid method. (B) High Performance Thin Layer Chromatography (HPTLC) of purified fractions for monosaccharides identification using sugar standards. HPTC plate was revealed by orcinol-sulfuric acid spray reagent. Std: standard; FA: Faction A; FB: Fraction B; Ram: rhamnose; Man: mannose; Glu: glucose; Gal: galactose.
